# Immunological Insights and Therapeutic Advances in COPD: Exploring Oral Bacterial Vaccines for Immune Modulation and Clinical Improvement

**DOI:** 10.3390/vaccines13020107

**Published:** 2025-01-22

**Authors:** Sławomir Lewicki, Barbara Joanna Bałan, Marta Stelmasiak, Dorota Magdalena Radomska-Leśniewska, Łukasz Szymański, Natalia Rios-Turek, Justyna Bień-Kalinowska, Łukasz Szarpak, Bogdan Hajduk

**Affiliations:** 1 Institute of Outcomes Research, Maria Sklodowska-Curie Medical Academy, Pl. Żelaznej Bramy 10, 00-136 Warsaw, Poland; justyna.bien-kalinowska@uczelniamedyczna.com.pl (J.B.-K.); b.hajduk1@vp.pl (B.H.); 2 Department of Environmental Threat Prevention, Allergology and Immunology, Faculty of Health Sciences, Medical University of Warsaw, Pawińskiego 3c, 02-106 Warsaw, Poland; 3 Department of Dietetics, Institute of Human Nutrition Science, Warsaw University of Life Sciences, Nowoursynowska 159c St., 02-776 Warsaw, Poland; marta_stelmasiak@sggw.edu.pl; 4 Department of Histology and Embryology, Medical University of Warsaw, Chałubińskiego 5, 02-004 Warsaw, Poland; dorota.radomska-lesniewska@wum.edu.pl; 5 Department of Molecular Biology, Institute of Genetics and Animal Biotechnology, Polish Academy of Sciences, Postępu 36A, 05-552 Magdalenka, Poland; l.szymanski@igbzpan.pl; 6 Hull University Teaching Hospitals NHS Trust, Hull University, Anlaby Rd., Hull HU3 2JZ, UK; gumita@vp.pl; 7 Institute of Medicine Science, Collegium Medicum, The John Paul II Catholic University of Lublin, 20-708 Lublin, Poland; lukasz.szarpak@kul.pl; 8 Department of Clinical Research and Development, LUXMED Group, 02-678 Warsaw, Poland; 9 Henry JN Taub Department of Emergency Medicine, Baylor College of Medicine, Houston, TX 77030, USA; 10 TS Out-Patients Clinic for Cardiovascular and Pulmonary Diseases, 01-460 Warsaw, Poland; b.hajduk1@vp.pl

**Keywords:** COPD, immune system, immunomodulation, bacterial vaccines

## Abstract

Chronic obstructive pulmonary disease (COPD) is a prevalent chronic condition associated with substantial global morbidity and mortality. Primarily caused by prolonged exposure to harmful agents such as dust and gases, COPD is characterized by persistent airflow limitation, clinically manifesting as chronic cough, sputum production, and dyspnea. The disease course alternates between stable phases and exacerbations, with the latter often associated with pathogenic colonization of the respiratory tract. This review examines the immunological underpinnings of COPD, emphasizing the interplay between innate and adaptive immunity in disease pathogenesis. Dysregulated immune responses to environmental factors perpetuate chronic inflammation, resulting in progressive pulmonary epithelial damage and connective tissue hyperplasia, which compromise gas exchange. Exacerbations further exacerbate respiratory failure, aggravating patient symptoms and accelerating disease progression. Despite advances in COPD management, effective therapeutic options remain limited. Current treatments primarily aim to alleviate symptoms, reduce immune activation, and manage infections, yet many patients experience suboptimal outcomes. This review highlights the potential of novel therapeutic approaches targeting immune system cells and pathways. In particular, it explores the promise of oral bacterial vaccines as immunomodulatory agents to enhance immune responses and improve clinical outcomes in COPD, addressing critical gaps in current treatment paradigms.

## 1. Introduction

Chronic obstructive pulmonary disease (COPD) is a leading global health concern, ranking as the third most common cause of death worldwide, following stroke and heart disease. In Poland alone, approximately 2 million individuals are affected by COPD, with over 250 million cases diagnosed globally. Despite its severe and progressive nature, COPD is both preventable and manageable; however, addressing its underlying causes remains a significant therapeutic challenge. The disease arises from a chronic and exaggerated inflammatory response to harmful environmental exposures, particularly particulate matter and gases—most notably from tobacco smoke and environmental pollutants—which leads to persistent and progressive airflow limitation in the respiratory tract [1]. Frequent exacerbations, commonly induced by infections and comorbid health conditions, further accelerate disease progression and worsen patient outcomes. A hallmark feature of COPD is excessive mucus production, which is universally present among patients and significantly affects clinical outcomes, including impaired lung function, diminished quality of life, increased frequency of exacerbations, higher hospitalization rates, and elevated overall mortality [2].

## 2. Characteristic of COPD

COPD primarily affects the lungs by obstructing airflow, and its main symptoms include cough, sputum production, and shortness of breath (dyspnea). Coughing is often an early symptom of COPD; it initially occurs intermittently, especially after respiratory infections, and tends to worsen during the autumn and winter months while subsiding in the summer [3]. A hallmark of COPD-related cough is its prevalence upon waking in the morning. As the disease progresses, the cough becomes chronic, occurring more frequently throughout the day and occasionally disrupting sleep. Initially dry, the cough often transitions to a productive one, with phases of dry and productive coughing alternating over time, especially during infections or colder months. Many patients describe this cough as mild, resembling throat-clearing, yet accompanied by sputum production. Consequently, some may deny having a cough, acknowledging only the act of clearing bronchial secretions. In severe cases, intense coughing may lead to syncope or even rib fractures. In the early stages of COPD, expectorated secretions are typically minimal, thin, and clear, but as the disease advances, they become more voluminous and muco-purulent or purulent [4]. Approximately 30% of COPD patients experience both coughing and the expectoration of significant amounts of sputum, with purulent secretions often indicating bronchiectasis. Other symptoms, such as wheezing, rhonchi during breathing, and chest tightness, may also occur. Wheezing, a whistling sound in the chest due to narrowed airways, tends to worsen in later stages of COPD, as do the other symptoms [5].

Dyspnea is the hallmark symptom of chronic obstructive pulmonary disease (COPD). In its early stages, patients may only experience shortness of breath or fatigue during intense physical activity. This reduced exercise tolerance results from irreversible airway obstruction, the destruction of alveolar spaces beyond the terminal bronchioles (emphysema), and air trapping [6]. As COPD advances, dyspnea can arise during routine activities, and some patients may even experience it at rest, either periodically or continuously. While some patients report dyspnea, others describe general fatigue or a persistent “lack of energy” instead [7]. In severe or very severe stages of COPD, patients may also experience symptoms such as loss of appetite, weight loss, reduced muscle mass, and mood disorders like depression or anxiety [8,9]. Right-sided heart failure (cor pulmonale) can cause edema in the lower extremities due to elevated pressure in the pulmonary artery.

The underlying mechanism of COPD involves the chronic activation of immune cells by irritants, with continuous release of inflammatory mediators. This chronic inflammatory state paradoxically reduces the immune system’s ability to fight infections, leading to persistent bacterial infections [10]. The exacerbations of COPD due to infections often occur without fever, instead presenting as worsened cough and dyspnea, along with a change in sputum from mucoid to purulent. COPD is thus characterized by a chronic inflammatory response in the airways that contributes to lung tissue damage and impaired lung function. Despite advances in understanding the mechanisms underlying COPD and identifying its causes, prevention and treatment strategies have remained largely unchanged. Increasing evidence suggests that the immune system plays a central role in COPD pathogenesis, with recurrent immune cell activation driving the chronic inflammatory response that characterizes the disease [11].

Although COPD primarily affects the lungs, it also has systemic consequences. COPD patients have an increased susceptibility to respiratory infections from viruses such as rhinoviruses (the most commonly detected viruses in COPD patients), influenza, and respiratory syncytial virus (RSV). Bacteria, including *Haemophilus influenzae*, *Streptococcus pneumoniae*, and *Moraxella catarrhalis*, are found in 10–30% of patients with stable COPD and in over 50% during exacerbations [12,13]. Other species of bacterial pathogens were also reported, of which *Pseudomonas aeruginosa* was the most predominant organism, followed by *Klebsiella pneumoniae*, *Staphylococcus aureus*, *Streptococcus pneumoniae*, and *Acinetobacter* spp. [14]. COPD patients also have an increased susceptibility to fungal colonization in the respiratory tract. The presence of colonization with *Aspergillus fumigatus* could cause significant changes in clinical manifestations (can aggravate airway hyperresponsiveness and induce sustainable airway inflammation and bronchoconstriction) and treatment outcomes for COPD patients [15]. Notably, the human respiratory tract, even without infection or obstructive disease, demonstrates remarkable diversity in its microbiome, which plays a protective role [16]. It is possible that specific interactions between hosts and microbes in the distal airways, influenced by bacterial colonization, contribute to COPD progression.

Three parasites migrate to the lungs at some point in their life cycle: *Ascaris lumbricoides*, *Strongyloides stercoralis*, and *Toxocara canis*. COPD patients with a parasitic infection may be susceptible to the development and exacerbation of COPD [17].

During infections, COPD patients’ clinical conditions typically deteriorate, reflecting progression in pathological changes and increasing disease severity.

## 3. Role of Immune System in COPD

**Figure 1:**
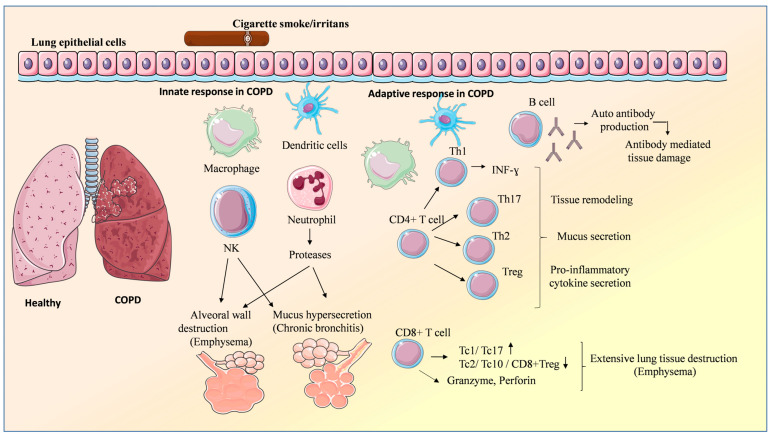
Innate and adaptive immune cells in the pathogenesis of chronic obstructive pulmonary disease (COPD). Risk factors (i.e., cigarette smoke) activate epithelial cells and innate immune cells such as macrophages, neutrophils, and natural killer cells. Activated dendritic cells orchestrate adaptive immune responses including CD8+ T cells, CD4+ T cells, and B-cell responses, leading to the development of chronic inflammation. ↑ increase, ↓ decrease.

The activation of both innate and adaptive immune mechanisms, generally characterized by an imbalance between pro-inflammatory and anti-inflammatory cells such as M1/M2 macrophages, Tc1/Tc10 lymphocytes, and Th17/Treg cells, drives the chronic inflammatory state in the pathogenesis of COPD. These cells release mediators that circulate in the peripheral blood and are also present in the lungs. Consequently, the primary abnormalities observed in the respiratory tract include the presence of inflammatory cellular infiltration resulting from leukocyte migration and uncontrolled cellular infiltration of lung tissue, as well as bronchial remodeling, which is characterized by the thickening of the airway walls [18]. Figure 1 and Table 1 summarize and detail the influence of individual components of the innate and adaptive immune systems.

**Table 1:** The role of innate and adaptive immune system in COPD pathogenesis. ↑ increase, ↓ decrease, rTreg (resting Treg), a Treg (activated Treg).

Cells Type	Lungs		
	Cell Frequency	Cell Phenotype/Subpopulations	Correlation with COPD Severity
Neutrophils	Increase	Proteases neutrophil elastase, cathepsin G, proteinase 3	Positive Alveoral wall destruction—emphysema; mucus hypersecretion—chronic bronchitis COPD severity
Macrophages	Increase	M2	Positive
Dendritic cells	Increase	-	Positive
NK cells	Increase	CD56 ↓	Positive Alveoral wall destruction—emphysema; mucus hypersecretion—chronic bronchitis COPD severity
		CD57 ↑	
		(CD69 + CD25-, CD69 + CD25+, CD69-CD25+)↑	
T cytotoxic CD8	Increase	Tc1cell/Tc17cell ↑	Positive Proliferation, apoptosis of pro-inflammatory CD8+ cell, mucus hypersecretion
		Tc2 cell/Tc10 cell/CD8+ Treg cell ↓	
		IFN-γ TNF-α ↑	
		IL-4 IL-13 ↑	
		Tc17 cell IL-17A, IL-17F ↑	
		Granzyme, Perforin ↑	
T helper Th17	Increase	↑ IL-17 → IL-1β, IL-6, G-CSF, GM-CSF, TNF-α, CXCL8	Positive Tissue remodeling
Treg cells		↑ rTreg, ↓ aTreg ↑Treg (FrIII)-	Positive Pro-inflammatory cytokine secretion
B cells	Increase	IgG B cell	Positive Antibody mediated tissue damage

### 3.1. Innate Immunity

The epithelial barrier of the respiratory tract houses various populations of epithelial cells, including ciliated cells (which produce mucus), endocrine cells, basal cells, and host defense cells of hematopoietic origin, such as granulocytes, mast cells, pulmonary macrophages, NK cells, and dendritic cells [19]. This system is constantly influenced by a range of exogenous (environmental factors) and endogenous factors, which together determine the state of homeostasis. Chronic exposure to environmental pollutants, such as tobacco smoke, and other harmful agents, along with endogenous processes occurring within the lungs, such as aging and pathological changes associated with past diseases, may reduce the defensive capabilities of the innate immune system in the lungs, leading to an increased susceptibility to bacterial and viral infections of the respiratory tract [20]. Primary infections elevate the risk of secondary infections, thereby contributing to lung tissue damage and the progressive deterioration of pulmonary function in patients with COPD. Additionally, the chronic inflammation and tissue damage observed in COPD further suppress the innate immune mechanisms in the lungs.

#### 3.1.1. Lung Epithelial Cells

The respiratory epithelium is a layer of epithelial cells lining the tracheobronchial tree, where it interacts with inhaled environmental pollutants, such as compounds in tobacco smoke, e-cigarette liquids, various toxins, and airborne pathogens. In a healthy state, the respiratory epithelium consists of ciliated cells and mucus-producing cells [21]. The primary role of the epithelium is to act as a physical barrier against external elements; it is reinforced by tight intercellular junctions that prevent microorganisms and xenobiotics from penetrating deeper tissues. Additionally, epithelial cells help remove foreign particles, including microorganisms, through mucociliary clearance. When activated, these cells can produce antimicrobial proteins like defensins, as well as pro- and anti-inflammatory cytokines, which contribute to the immunoregulatory and protective functions of the respiratory epithelium [22].

Exposure to environmental pollutants, repeated respiratory infections, and cigarette smoke can cause destructive changes to the respiratory epithelium’s structure. Pathological squamous cells replace differentiated, functional secretory, and ciliated cells in squamous metaplasia, and mucous cell hyperplasia leads to excessive mucus secretion into the airway lumen, resulting in its subsequent obstruction [23]. Damage to the ciliated epithelium, through a reduction in ciliated cell numbers, the shortening of cilia, and disrupting their rhythmic movement, decreases the efficacy of mucociliary clearance of respiratory pathogens [24]. This damage is accompanied by a reduced expression of receptors for the secretory component of secretory immunoglobulin A (sIgA), which transports it to the mucosal surface, as well as by the inhibition of nuclear factor (NF)-κB transcriptional activation and interferon production in response to viral infection [25]. The ensuing inflammatory state contributes to increased epithelial permeability in the airways.

#### 3.1.2. Natural Killer (NK) Cells

Numerous studies indicate that natural killer (NK) cells play a crucial role in the pathogenesis of lung diseases, including COPD. Research by Osterburg et al., conducted across five patient groups—healthy smokers (HS), healthy nonsmokers (HN), former smokers (FS), and COPD patients classified as GOLD I/II and GOLD III/IV—revealed that COPD patients and healthy smokers exhibited distinctive NK cell populations and displayed enhanced cytotoxicity and reduced cytokine responsiveness [26]. The authors suggest that chronic, tobacco-driven inflammation may promote the activation and maturation of NK cells. They hypothesize that the observed reduction in CD57+ cells in the blood with advancing disease severity may be due to NK cell migration to inflamed lung tissues. Supporting these findings, other authors showed increased densities of CD57-enriched NK cells within pulmonary tissue and lymphoid follicles in lungs of severe COPD patients, suggesting that, following inflammatory stimulation as seen in COPD, NK cells migrate from the bloodstream to the lungs, where they may contribute to COPD-related pathologies [27,28]. Moreover, Brajer-Luftmann et al. showed that the peripheral blood and BALF of COPD patients contained more immature NK cells (CD11b- phenotype) with regulatory function compared to the HN control group, a finding that could potentially link the general and local inflammatory states of COPD patients [28].

In COPD, the quantity and functionality of activating and inhibitory NK cell receptors undergo significant alterations. Overall, the expression of NK cell-activating receptors generally decreases while inhibitory receptor expression increases [28]. Moreover, Wang et al. identified a strong association between NK cell-activating receptors and both COPD stage and smoking status [29]. The proportion of activated NK cells (CD69+) increased in the peripheral blood and induced sputum among healthy smokers, former smokers with COPD, and current smokers, as compared to healthy nonsmokers. However, other studies, including Hodge et al. and Pascual-Guardia et al., did not find a significant relationship between peripheral blood NK cells expressing the CD69+ phenotype and disease severity, smoking status, or exacerbation frequency in COPD patients [30,31]. Peripheral blood NK cells in COPD patients also exhibited also increased expression of regulatory receptors CD158b, CD158i, and CD314 (NKG2D). CD158, with both activating and inhibitory potential, modulates NK cell activity via MHC I interactions. Brajer-Luftmann et al. found correlations between NK cell receptors CD158b+, CD314+, and CD336+ (involved in cytotoxic response activation) with age, pulmonary function parameters (RV, TLC), smoking history, and exacerbation frequency in COPD patients [28]. Additionally, autologous pulmonary epithelial cells may increase the expression of MICA/MICB ligands for NKG2D activation [32]. These findings align with studies by Tang et al., which described increased expression of inhibitory receptors CD158a+ and CD158b+ in peripheral NK cells of COPD patients, negatively correlating with lung function [33]. Some studies have shown a decrease in the frequency of NK cells expressing CD158e1 in the peripheral blood among healthy smokers and COPD smokers compared to healthy nonsmokers [29], along with reduced expression of the inhibitory CD94 receptor on peripheral NK cells and BALF NK cells in COPD smokers relative to nonsmokers [30]. While these findings suggest a pivotal role for NK cells in COPD pathogenesis, further research is required to clarify the inconsistent results.

Findings regarding direct NK cell cytotoxicity in COPD patients compared to healthy smokers (HS) and healthy nonsmokers (HN) have been inconsistent. Hodge et al. observed heightened NK cytotoxicity in BALF samples from COPD patients compared to HS and HN [30]. Moreover, studies by Finch et al. suggested that increased airway obstruction, as measured by predicted FEV1, could trigger an increase in NK cell cytotoxicity [34]. Other studies reported elevated lung NK cell cytotoxicity, which is positively correlated with stress-induced responses and emphysema progression [32]. Conversely, Urbanowicz et al. identified a decrease in direct periherial NK cytotoxicity in COPD patients compared to HS and HN, correlating with worsening lung function [35]. Studies on perforin and granzyme production by NK cells in COPD patients also yield contradictory results. Hodge et al. reported an increased proportion of NK cells producing granzyme B in COPD patients’ peripheral blood and BALF compared to HS [30]. Urbanowicz et al. also showed a higher proportion of periherial NK cells producing perforin, yet not granzyme, in COPD patients compared to HS and HN [35]. Moreover, these authors observed elevated perforin- and granzyme B-producing cells in induced sputum from COPD patients compared to HS and HN. Contrastingly, Tang et al. found a reduced proportion of peripheral NK cells expressing perforin and granzyme B in COPD patients compared to HS and HN, alongside a reduction in IFN-γ production in COPD patients relative to HN [32].

#### 3.1.3. Neutrophils

Airway neutrophilia is a hallmark of various lung diseases, including COPD. Factors such as tobacco smoke, infectious agents, and inflammatory lung conditions drive this phenomenon. Dysregulated neutrophil numbers or function can contribute to lung tissue damage as these cells infiltrate lung tissue [36]. Neutrophil migration is notably impaired in COPD, resulting in an elevated lung neutrophil count compared to healthy individuals due to increased migration [37]. The migration process begins with changes in cell polarization; neutrophils from COPD patients exhibit more frequent spontaneous polarization and less directional polarization than those from healthy smokers and non-smokers [38]. Moreover, while neutrophils in COPD demonstrate accelerated migration, their accuracy in moving toward specific chemoattractants is reduced [39]. During migration, neutrophils release serine proteases such as neutrophil elastase (NE), cathepsin G, and proteinase 3 (PR3) from azurophilic granules into the extracellular space [40]. Neutrophils from COPD patients show increased degranulation, with upregulated surface expression of the primary granule marker CD63 on unstimulated neutrophils [41]. These proteases, though crucial for pathogen clearance, can also damage host tissues. The dysregulation of the protease/anti-protease balance in COPD results in lung tissue damage [42]. Neutrophil-derived serine proteases exacerbate airway pathology by increasing mucus secretion from goblet cells and submucosal glands, contributing to airway obstruction and a heightened risk of bacterial infections [43]. Additionally, studies by Genschmer et al. reveal that neutrophils in COPD release exosomes that bind neutrophil elastase, allowing them to persist in tissues and resist inhibition by the AAT inhibitor. These neutrophil elastase-bound exosomes attach to the extracellular matrix (via Mac-1), sustaining proteolytic activity against matrix proteins. Researchers showed that NE-loaded exosomes transfer a COPD-like phenotype in a murine model [44].

COPD patients’ sputum also exhibits increased components of neutrophil extracellular traps (NETs) and a higher number of NET-producing neutrophils [45]. Researchers have found a link between the sputum microbiome and NET formation, which correlates with COPD severity and FEV1 levels. In regions where *Haemophilus* species predominate, 40% of these bacteria are associated with significantly larger DNA-elastase complexes, which may enhance tissue damage [46]. However, contrary findings by Hakkim et al. showed that neutrophils isolated from sputum during COPD exacerbations had a reduced capacity for NET formation compared to those from stable COPD patients or healthy controls [47]. While phagocytosis is the primary means by which neutrophils target pathogens, few studies have explored this function in COPD. Results are inconsistent, with some studies showing no difference in phagocytic activity compared to healthy individuals and others suggesting impaired phagocytosis [48]. Decreased phagocytosis has been documented in response to pathogens such as *Haemophilus influenzae*, *Streptococcus pneumoniae*, and apoptotic neutrophils via efferocytosis [49,50,51]. Disorders in phagocytosis and prolong lung inflammation reduce the ability of the tissues to regenerate and cause their constant remodeling.

Overall, neutrophils play a central role in COPD pathology. The altered neutrophil functions seen in COPD, including increased protease release, accumulation in the lungs, and the emergence of distinct cellular phenotypes, collectively contribute to lung tissue damage and disease progression.

### 3.2. Adaptive Immunity

Cells of the adaptive immune system play a critical role in eliminating exogenous threats. However, the excessive activation or modulation of these cells can induce changes that, due to the mechanisms of adaptive immunity, are difficult to reverse spontaneously. Researchers have also described the negative role of adaptive immune cells in the development, persistence, and progression of COPD. Among these cells, key players include helper T cells (CD4+), cytotoxic T cells (CD8+), and B lymphocytes. Below, we explore how these cells contribute to chronic inflammation and the remodeling of the pulmonary epithelium.

#### 3.2.1. CD8+ T Cells

A significant increase in cytotoxic CD8+ T cell counts within the lungs has been consistently observed in COPD, including in lung tissue biopsies [52], bronchoalveolar lavage fluid [53], induced sputum [54], and bronchial mucosa [55]. Evidence suggests that CD8+ cell numbers in the lungs may correlate with disease severity. However, most studies report elevated peripheral CD8+ levels in COPD patients [56,57], while others find no change [58] or even a decrease compared to controls [59]. Notable changes have been observed in specific T-cell subpopulations such as an increase in pro-inflammatory Tc1 and Tc17 cells, particularly during exacerbations, and a reduction in regulatory subtypes like Tc2, Tc10, and CD8+ Treg cells [57] compared to healthy non-smokers. Tc10 and CD8+ Treg cells play essential roles in controlling and suppressing the inflammation. Moreover, COPD has a strong association with cigarette smoking. While studies on the impact of tobacco smoke on CD8+ lymphocyte levels in peripheral blood have yielded inconclusive results regarding the exact effect on cell counts, smokers with COPD consistently show an increase in pro-inflammatory CD8+ cells and a decrease in CD8+ regulatory T cells. This shift correlates with enhanced proliferation and reduced apoptosis of pro-inflammatory CD8+ cells in response to cigarette smoke exposure [60].

In addition to changes in CD8+ cell numbers, COPD also involves functional alterations in these cells. Lung biopsies from COPD patients reveal increased production of pro-inflammatory cytokines, such as IFN-γ and TNF-α, by CD8+ T lymphocytes [61], with similar findings in the peripheral blood [62]. Additionally, some studies report elevated levels of CD8+ cells secreting IL-4 and IL-13 [63], as well as Tc17 cells producing IL-17A and IL-17F in lung tissue [64]. CD8+ cells isolated from COPD patients also exhibit increased expression of granzymes and perforin, which contribute to targeted cell apoptosis [65,66]. The extensive lung tissue destruction observed in emphysema could thus be a result of CD8+ lymphocyte activity. Moreover, CD8+ cells demonstrate elevated expression of FAS receptors, which can lead to an increased induction of apoptosis in adjacent cells. This state does not allow for proper cell regeneration and drives inflammation. A positive feedback mechanism that increases the influx of CD8+ cells into the lungs in COPD has also been described; CD8+ cells in the lungs release IFN-γ, promoting the production of the chemokine CXCL10, which enhances the infiltration of CXCR3 receptor-bearing cells (of the Th1 phenotype), thereby amplifying inflammatory and destructive activity while attracting additional pro-inflammatory cells [67].

#### 3.2.2. CD4+ T Cells

The balance between the pro-inflammatory response primarily generated by Th1 cells and the anti-inflammatory response mainly driven by Th2 lymphocytes is essential for maintaining homeostasis within the cellular and humoral immune responses. In the lungs of COPD patients, this balance shifts toward Th1 dominance, resulting in chronic inflammation that ultimately leads to airway epithelial remodeling and emphysema [68]. The number of Th1 phenotype cells increases with disease progression, along with elevated IFN-γ production [69]. Another population that drives inflammation in the lungs is the Th17 cell population. Researchers have observed increased numbers of these cells in the bronchial submucosa, airway epithelium, lung tissue, bronchoalveolar lavage fluid, and peripheral blood of COPD patients [70,71]. Animal models have also confirmed the role of Th17 cells in COPD pathogenesis [72]. By secreting IL-17, Th17 cells influence the secretion of cytokines and chemokines that recruit neutrophils and macrophages, such as IL-1β, IL-6, G-CSF, GM-CSF, TNF-α, and CXCL8, contributing to tissue remodeling [73].

During acute exacerbations of COPD, there is an increase in the proportion of Th2 cells, characterized by predominant IL-4 secretion [74]. Th2-related responses are associated with the development of systemic sclerosis and fibrosing alveolitis. Additionally, cytokines secreted by Th2 lymphocytes (IL-4 and IL-13) lead to excessive mucus production, contributing to restricted airflow [75]. Treg lymphocytes should modulate the excessive activation of the Th1/Th17 pro-inflammatory or Th2 anti-inflammatory pathways. Unfortunately, a substantial reduction in Treg cell populations has been observed in both lung tissue and the peripheral blood of COPD patients [76,77]. The balance among Treg subsets is also altered. In humans, Treg cells are thought to comprise three distinct subsets: resting Treg (rTreg), activated suppressive Treg (aTreg), and cytokine-secreting pro-inflammatory Treg (Fr III). In COPD patients, a decrease in rTreg and aTreg populations, alongside an increase in Fr III cells, has been noted [78]. Changes in Treg cells may further promote pro-inflammatory cytokine secretion, which suppresses FoxP3 expression, inhibits Treg differentiation, and contributes to COPD progression. Some authors also report impaired Treg function within the lung microenvironment [79].

#### 3.2.3. B-Cells

Researchers have observed an increase in both B-cell numbers and lymphoid follicles rich in B lymphocytes in the lungs of COPD patients [80]. The increase in B-cell infiltration and the number of lymphoid follicles correlate with disease severity. The chemokine CXCL13 is responsible for the induction of lymphoid follicle formation in the lungs, operating through a positive feedback loop. CXCL13 is actively secreted by B lymphocytes isolated from the lungs of COPD patients [81] as well as asthma patients [82]. This chemokine promotes increased migration of B cells to the lungs and enhances lymphotoxin expression. Lymphotoxin supports the de novo formation of lymphoid follicles and further stimulates CXCL13 production through B lymphocytes [83]. Additionally, B-cell survival in the lymphoid follicles of COPD patients’ lungs is bolstered by the increased secretion of factors such as SDF-1, CXCL12, a proliferation-inducing ligand (APRIL), and B-cell activating factor (BAFF) [84,85]. Interestingly, in COPD patients, BAFF also enhances the survival of CD8+ lymphocytes [86]. The accumulation of B lymphocytes in COPD patients’ lungs, along with chronic inflammation, produces autoantibodies targeting lung cells, cellular components, and extracellular matrix proteins. These autoantibodies sustain lung inflammation by directly or indirectly activating the complement pathway through immune complex formation.

Various adaptive immune system cells contribute to COPD pathogenesis and progression. Treatment strategies targeting a single lymphocyte population have not achieved sufficient therapeutic responses. For instance, increased lung infections in the rituximab arm prematurely halted a randomized, controlled clinical trial using rituximab (a monoclonal antibody that induces apoptosis in human B cells and reduces activated B-cell numbers) [87]. However, a recently published clinical study indicated that rituximab was more effective in alleviating symptoms in patients with connective tissue disease-associated interstitial lung disease in the UK compared to cyclophosphamide [88]. The common immunological feature of connective tissue disease-related interstitial lung disease and COPD is chronic inflammation, which plays a key role in lung structural damage, although through various mechanisms (autoimmune vs. exposure to external factors such as tobacco smoke). These findings suggest that systemic immunosuppressive therapy may be a suboptimal approach for COPD treatment. Instead, the specific modulation of immune system activity, aimed at regulating all adaptive immune cell types involved in COPD pathogenesis, may provide a more balanced approach. Therapeutic strategies focusing on immune modulation, rather than broad immunosuppression, could help reduce inflammation without compromising the body’s ability to defend against infections.

## 4. Standard and Alternative Methods of COPD Treatment

COPD is characterized by the broad suppression of the innate immune system, making it essential to implement preventive strategies that restore the disrupted components of innate immunity. Given that multiple elements of innate immunity are compromised in COPD, likely as part of a common pathological process triggered by environmental pollutants, future therapies should aim to address these multiple disruptions simultaneously by targeting shared pathological mechanisms [89]. Smoking cessation should certainly be considered as a primary approach as most altered innate immune features in COPD result from the immunosuppressive effects of cigarette smoke. For COPD patients with additional risk factors that may modify innate immune mechanisms aside from or independently of smoking, such as occupational pollutant exposure, alcoholism, obesity, chronic kidney disease, insufficient physical activity, and altered micronutrient levels, targeted actions addressing these cofactors of disease pathogenesis should be considered as fundamental therapeutic strategies [90]. Furthermore, certain COPD treatments, including glucocorticosteroids, oxygen supplementation, and methylxanthines, may suppress aspects of innate immunity. Therefore, therapeutic strategies aimed at bolstering the innate defense mechanisms in COPD should also consider both disease-related immune alterations and potential immune suppression induced by standard therapies [91].

Therapeutic approaches to enhance defense mechanisms and modulate mucosal and systemic immune responses in COPD have been explored extensively. Below, we outline several biological strategies for the treatment of COPD, as summarized in Table 2. These approaches focus on targeting key pathological mechanisms, including inflammation, mucus overproduction, and immune dysregulation, aiming to improve clinical outcomes and patient quality of life.

### 4.1. Inhibitors

Epidermal growth factor receptor (EGFR) signaling disrupts the integrity of the respiratory epithelial barrier, and airway-targeted therapies that locally control EGFR activity may help restore epithelial defenses in smokers with COPD. However, the use of BIBW 2948 BS, an inhaled EGFR inhibitor, did not significantly reduce epithelial mucin stores and was poorly tolerated by patients with COPD [92]. Physical exercise, on the other hand, may aid in clearing excess mucus from the airways, thereby supporting the mucociliary barrier’s function [93,94]. While these strategies show promise in improving epithelial barrier function and enhancing mucosal defenses, they have yet to deliver a transformative impact on COPD treatment, underscoring the need for innovative therapeutic approaches that more effectively mitigate disease progression and reduce exacerbation frequency. One such approach involves the use of roflumilast, which exerts anti-inflammatory effects by reducing the release of inflammatory mediators from neutrophils and decreasing pro-inflammatory cytokine production. Another promising strategy targets phosphoinositide 3-kinase (PI3K), a pivotal mediator of pathological airway changes in smokers. PI3K inhibition has been shown to restore innate immune functions such as neutrophil chemotaxis in COPD patients. Specifically, the PI3K inhibitor IC-87114 has demonstrated efficacy in reducing neutrophil recruitment and restoring corticosteroid sensitivity in human COPD patients [95]. Furthermore, the PI3K inhibitor LY 294,002 has been shown to reduce monocyte/macrophage adhesion and infiltration by decreasing the expression of an intercellular adhesion molecule (ICAM) in COPD patients [96]. Another PI3K inhibitor, NVS-PI3K (variants no. 2, 3, and 5), has demonstrated efficacy in reducing lung inflammation and bacterial colonization without negatively impacting alveolar macrophage phagocytosis in COPD patients [50]. Additionally, therapeutic overexpression of elafin, an antimicrobial peptide and elastase inhibitor derived from epithelial and innate immune cells, may protect against emphysematous lung damage from neutrophil elastase while enhancing host defenses by activating pulmonary dendritic cells [97].

Finally, zinc supplementation, which has shown potential in improving NK cell function and reducing systemic inflammation in elderly individuals, could also be useful in normalizing disrupted innate immunity in COPD.

### 4.2. Antibodies

COPD is predominantly driven by a Th1-dependent immune response characterized by neutrophilic infiltration. This inflammatory cascade is mediated by pro-inflammatory cytokines, including TNF-α, IL-1, IL-6, IL-8, and IL-17, among others. While anti-TNF-α antibodies, such as infliximab, have been evaluated for their therapeutic potential, studies revealed no significant benefits on sputum neutrophils, lung function, resting energy expenditure, or quality of life. Moreover, the treatment was associated with an increased risk of adverse events, including pneumonia and cancer [98]. Similarly, the use of anti-IL-6 antibodies in COPD has not demonstrated clear efficacy. IL-8, a critical cytokine mediating neutrophil and monocyte chemotaxis, degranulation, and activation, has shown promise in targeted approaches. Both blocking antibodies (ABX) and receptor antagonists (MK-7123/navarixin) have demonstrated positive effects in some COPD patients [99,100].

In contrast, a subset of COPD patients exhibits a Th2-dependent immune response with eosinophilic infiltration. Cytokines mediating this type of response include IL-25, TSLP, IL-4, IL-5, IL-9, and IL-13 [101]. Among these, IL-33 plays a pivotal role in the secretion of Th2 cytokines. By binding to the ST2 receptor on ILC2, mast cells, NK cells, and endothelial cells, IL-33 triggers a cascade of type 2 inflammatory responses [102]. It also influences endothelial and lung epithelial cells to secrete IL-6 and IL-8, which enhance neutrophil migration. Clinical trials are ongoing for antibodies targeting IL-33’s effector actions, including itepekimab, astegolimab, and tozorakimab [103,104,105]. Other strategies targeting Th2-associated cytokines include IL-5 (mepolizumab), IL-5R (benralizumab), and IL-4/IL-13 receptor antagonists (dupilumab) [106,107,108]. However, clinical trials for IL-13 inhibitors (lebrikizumab and tralokinumab) and thymic stromal lymphopoietin (TSLP) inhibitors (tezepelumab) have yet to provide conclusive evidence regarding their efficacy in COPD [109,110].

B-cell-targeting therapies have also been explored. Rituximab, which targets CD20, showed potential but was associated with an increased risk of pulmonary infections in COPD patients. Belimumab, targeting the B-cell activating factor, is another potential candidate, though its utility remains under investigation [111].

Despite advances in anti-inflammatory and immunomodulatory therapies focusing on adhesion molecules, cytokine signaling, and lipid metabolism, their capacity to enhance specific components of innate immunity in COPD remains uncertain [50]. Unfortunately, the treatment options explored so far have not provided satisfactory results, as persistent inflammation in COPD leads to ongoing damage to the respiratory epithelium and, with disease progression, more frequent and severe exacerbations. This unmet need is widely recognized as evidenced by numerous clinical trials currently underway to investigate new drugs and treatment regimens for COPD [112].

**Table 2:** Biological strategies for COPD treatment.

**Inhibitors**		
Roflumilast	PDE4 inhibitor, reduces exacerbations and hospitalizations in severe COPD.	[113,114]
IC-87114	PI3K inhibitor, reduces neutrophils recruitment, restores corticosteroid sensitivity	[95]
LY 294002	PI3K inhibitor, inhibits the expression of intercellular adhesion molecule−1(ICAM-1) in COPD patients, mediating monocyte/macrophage adhesion and infiltrating	[96]
NVS-PI3K-2, -3, -5	PI3K inhibitor, suppresses lung inflammation and bacterial colonization in COPD patients.	[50]
Elafin	Serine protease inhibitor, offers protection against emphysematous lung damage from neutrophil elastase, enhancing host defenses by activating pulmonary dendritic cell	[97]
MK-7123/navarixin	IL-8 receptor antagonist, improvement in forced expiratory volume in 1	[100]
**Antibodies**		
Abx	Blocking IL-8 results in a significant improvement in dyspnea, measured using the transitional dyspnea index	[99]
Itepekimab	Blocking IL-33 reduces inflammation of the airways	[103]
Astegolimab	Blocking IL-33 binding to the ST2 reduces exacerbations in patients with moderate to very severe COPD	[104]
Tozorakimab	Blocking IL-33 binding to the ST2 receptor causes a decrease in excess inflammation and epithelial remodeling in diseases caused by IL-33	[105]
Mepolizumab	Blocking IL-5 reduces the frequency of moderate and severe exacerbations and extends the time between subsequent exacerbations	[106]
Benralizumab	Blocking the alpha subunit of the interleukin 5 receptor reduces the risk of moderate or severe COPD exacerbation within 30 or 90 days	[107]
Dupilumab	Blocking IL-4/13 receptor results in fewer exacerbations, better lung function and quality of life, and less severe respiratory symptoms	[108]
Belimumab	Blocking B-cell activating factor (BAFF) reduces autoantibody anti-GRP78 levels	[111]

## 5. Potential of Oral Bacterial Vaccines

In the 19th and 20th centuries, the use of vaccinations resulted in a significant reduction in the incidence of certain infectious diseases, even eliminating some of them. The World Health Organization (WHO) estimates that vaccinations prevent an average of 2–3 million deaths yearly. Significantly more people are protected from acute illnesses and long-term disabilities from infectious diseases. Vaccination is, therefore, one of the most effective and cost-effective public health interventions [115]. Vaccination induces a reaction like natural contact with an antigen. The effect of such an intervention is the initial activation of innate mechanisms (inflammatory infiltration), and then acquired mechanisms involving T and B lymphocytes and the antibodies they produce.

Currently, there are many types of vaccines, both traditional and innovative [25]. Typical routes of vaccine administration to induce a systemic immune response are intramuscular and intradermal. Mucosal vaccines are also used. The surface of the mucosa is on average 200 times larger than the skin surface and is a site of penetration for many pathogens and allergens [30]. Targeting vaccine administration through the mucosa to induce local and systemic immunity is a new direction in preventing and controlling many infectious diseases. The protective effect of bacterial immunostimulants is attributed to the enhancement of non-specific immunity, as well as the general activation of cellular and humoral responses [116].

The use of bacterial lysates as immunostimulatory agents in the treatment of COPD has been the subject of extensive debate and evaluation across numerous clinical trials. Additionally, meta-analyses conducted on large patient cohorts have provided compelling evidence supporting the protective effects of bacterial lysates. These studies demonstrate that bacterial lysates significantly reduce respiratory infection-related symptoms, decrease the frequency and severity of acute exacerbations, and reduce the duration and number of hospitalizations. Furthermore, patients undergoing bacterial lysate therapy reported symptomatic relief and notable improvements in their quality of life. Table 3 summarizes key findings from the literature on the therapeutic effects of bacterial lysates in COPD, highlighting their potential role in complementing standard treatments and addressing the unmet needs in the management of this condition.

The gastrointestinal mucosa is also the most desirable and accepted route of administration of medicinal products by patients. Moreover, as indicated by results from observations, the oral route enables the stimulation of humoral and cellular immune responses, not only in mucosal sites but also systemically by stimulating the production of IgG and sIgA antibodies.

Oral administration of the vaccine stimulates CD4, CD8, and CD19 lymphocytes, the production of IL-1, 4, 6, 8, 10, 12, 13, and 17 cytokines, and IFN-γ; regulates the Th1/Th2 lymphocyte ratio; increases the number and activates neutrophils and macrophage phagocytosis; regulates the function of dendritic cells and M cells responsible for antigen presentation; and affects cell adhesion processes and the expression of HLA I and II molecules, as well as TLR4 and TLR9 receptors [126,127]. This has a comprehensive effect on the activation of the immune system, leading to reduced susceptibility to infections, including respiratory tract infections. Therefore, immunotherapy using oral bacterial vaccines is emerging as a promising approach for stabilizing COPD patients. This treatment modulates immune responses within the respiratory mucosa, simultaneously reducing inflammation and enhancing resistance to infections [117,128,129]. The normalization of lung epithelial dysfunction results in a significant clinical improvement in treating asthma, respiratory infections (including COVID-19), and COPD [130,131,132,133]. It is noteworthy that mucous membranes, beyond acting as physical–chemical barriers, are equipped with diverse innate and adaptive immune mechanisms due to resident immune cells, such as macrophages, dendritic cells, and lymphocytes. The mechanism of action of oral bacterial vaccines relies on mucosa-associated lymphoid tissue (MALT). When lymphocytes encounter an antigen at a mucosal site, these immune cells (e.g., T and B lymphocytes and dendritic cells), along with IgA and IgG antibodies, migrate to other mucosal surfaces, establishing systemic immunity [134].

The stimulation of MALT with bacterial antigens confers immunity against pathogens in the vaccines and contributes to the development and modulation of immune cell function. Oral vaccines that support the immune system primarily contain bacterial antigens from species that frequently cause respiratory infections. These vaccines not only induce specific immunity against these pathogens but also activate various defense mechanisms within MALT that may enhance nonspecific defenses against other pathogens, potentially including antiviral immunity. Antiviral immunity following the administration of bacterial lysates is largely associated with macrophage activation, proliferation, the stimulation of phagocytosis, and increased Toll-like receptor expression on lymphocytes, along with the production of IgG, secretory IgA (sIgA), and increased antibody-dependent cellular cytotoxicity (ADCC). Through the migration of IgA-producing cells, mucosal immunization leads to antigen-specific IgA presence at distant mucosal sites [135,136]. Additionally, the presence of adjuvants in vaccine preparations facilitates the capture of soluble antigens during mucosal delivery routes, promoting preferential uptake by antigen-presenting cells (APCs) and epithelial cells to stimulate specific immunity (sIgA), as well as cytotoxic and helper T-cell responses. IgA antibodies coat bacteria to prevent pathogen colonization of the mucosal epithelium. Moreover, sIgA neutralizes bacterial and viral protein toxins and may activate cells capable of spontaneous or antibody-dependent cytotoxicity (NK and CTLs), which enhances infection protection.

Oral vaccines influence lymphocytes by stimulating their activation, proliferation, and immune function enhancement, as well as restoring and maintaining the Th1/Th2 and CD4/CD8 balance [137]. The oral route offers a distinct advantage due to its convenience, high patient acceptance, and low incidence of adverse effects [138]. Given that both infection and inflammation are key drivers in COPD progression, therapies addressing these elements in COPD pathogenesis may offer particular benefits.

Currently, available nonspecific bacterial vaccines with immunomodulatory effects vary in composition and intended use. These oral preparations are made from bacterial lysates and contain antigens from pathogens most associated with infections in specific sites, namely, Broncho-Vaxom for respiratory infections, Urovaxom for urogenital infections, and vaccines like Dukoral and Vivotif for cholera and typhoid fever, respectively. These formulations typically include bacterial lysates, ribosomal fractions, and cell wall components from strains most frequently responsible for infections. However, the dosage and strain selection differ among products. For instance, each Broncho-Vaxom capsule contains either 7 mg or 3.5 mg (for children) of bacterial lysate derived from species such as *Staphylococcus aureus*, *Streptococcus viridans*, *Streptococcus pneumoniae* (multiple serotypes), *Streptococcus pyogenes*, *Klebsiella pneumoniae*, *Klebsiella ozaenae*, *Moraxella catarrhalis*, and *Haemophilus influenzae*. In contrast, Ismigen contains 7 mg of bacterial lysate (6 billion CFU) along with 43 mg of glycine, while Luivac includes 3 mg of bacterial lysate, and Urovaxom provides 60 mg of lyophilized *Escherichia coli*. These products are available in various forms, such as capsules, tablets, and sublingual tablets, providing options for different patient preferences and therapeutic needs.

Numerous studies have examined the effects of oral vaccines on immunity improvement, elucidating mechanisms involving monocyte and NK cell activation, T-cell activation (Th1, Treg, Tc), the expression of antimicrobial peptides in the epithelium and mucosa, macrophage activation, and the migration of polymorphic neutrophils to the lungs [139]. Recent findings suggest that oral vaccines may induce long-term changes in the gut microbiota, which may in turn affect the composition of respiratory tract microbiota [140]. Additionally, gut and nasopharyngeal microbiota profiles have been shown to have prognostic significance in COPD, correlating with the frequency of exacerbations [141]. Previous research has demonstrated that oral vaccine therapy can reduce the incidence of infections in recurrent respiratory infections, suggesting that for COPD patients, vaccine use may also decrease the frequency of exacerbation-triggering infections [142]. Results from a phase IV clinical trial assessing the efficacy of oral bacterial vaccines in COPD showed mainly prolonged effects of oral vaccination. There were no significant differences in the time of the first exacerbation compared to the placebo; however, the time to the second exacerbation was nearly doubled (median 103.5 vs. 60.5 days). This is attributed to the biological nature of the treatment, noting that unlike pharmacological drugs, oral bacterial vaccines require a longer period to take effect [129]. Similar observations have been reported in our clinical practice, where patients with advanced COPD (stages 3–4) who did not respond adequately to pharmacological treatments benefited from a regimen of Broncho-Vaxom, showing a substantial reduction in exacerbation frequency over the long term. Thus, long-term assessment of these preparations appears to be the most appropriate approach for evaluating their effectiveness in COPD management.

## 6. Conclusions

In summary, environmental pollutants, tobacco exposure, and the suppression or premature aging of both innate and adaptive immune components shape COPD, a chronic lung disease. This immune dysfunction likely contributes to increased susceptibility to respiratory infections and the development of secondary inflammatory conditions, both of which drive disease progression. Therefore, effective COPD management should extend beyond conventional anti-inflammatory treatments to include therapeutic strategies aimed at restoring normal immune function in both the innate and adaptive systems. Personalized treatments should address the specific cellular and molecular immune alterations unique to each patient, given the substantial clinical and biological heterogeneity within COPD. To this end, studies are needed to evaluate the efficacy of immunotherapies that incorporate antigens from bacteria most commonly associated with respiratory infections, aiming to provide targeted, immune-based support in COPD management.

## Figures and Tables

**Table 3:** Effects of bacterial lysate therapy in COPD.

The Described Effect of Including Oral Vaccines in the Treatment of CODP	Clinical Trials Confirming Beneficial Effects
Decrease in the incidence of acute exacerbations	[117,118,119]
No decrease in severe exacerbations	[120,121]
Delay in moderate COPD exacerbations	[120]
Reducing the incidence of respiratory tract infections	[117,122]
Significant reduction in the total number of exacerbations	[117,122,123]
Decreased in the need for antibiotics	[119,122,123]
Improve quality of life	[124]
Decrease in the need of hospitalization	[121]
Improve quality of life	[124]
Improve lung function	[123,124,125]
Improve lung function	[117,121,124,125]
Reduce number of hospitalizations	[117,119]

## Data Availability

Not applicable.
